# A novel outpatient treatment model for patients with severe and enduring anorexia nervosa: an observational study of patient characteristics, treatment goals, and treatment course

**DOI:** 10.1186/s40337-023-00877-x

**Published:** 2023-09-06

**Authors:** Monica Ålgars, Svetlana Oshukova, Jaana Suokas

**Affiliations:** 1 Eating Disorder Unit, HUS Helsinki University Hospital, PB 282, 00029 Helsinki, Finland; 2 Department of Psychology, University of Helsinki, PB 21, 00014 Helsinki, Finland

**Keywords:** Anorexia nervosa, Eating disorders, Treatment, Severe and enduring anorexia nervosa

## Abstract

**Background:**

Approximately 20–30% of people with anorexia nervosa develop an enduring form of the disorder. In the present study a newly developed outpatient treatment unit for patients with severe and enduring anorexia nervosa was described. The treatment model is flexible, patient-centered, and aims at enhancing quality of life, maintaining medical stability, and minimizing harm. Treatment contents, patient characteristics, treatment goals, and course of treatment from the first five years of operation were described and analyzed.

**Methods:**

The participants (*N* = 22) consisted of all referrals resulting in an assessment or treatment period at the unit between May 2017 and May 2022. All participants were women. The study was a registry study. Information regarding patient characteristics, treatment goals, and the course of treatment was gathered from medical records.

**Results:**

On average, the participants had had a diagnosed eating disorder for 12.80 years, and self-reported eating disorder symptoms for 19 years. Their symptomatology included severe eating disorder symptoms, psychiatric comorbidities, extreme underweight, and co-occurring medical conditions. Their treatment goals commonly concerned improving physical health, reducing eating disorder symptoms, improving psychological well-being, and improving quality of life. The majority of participants for whom this information was available benefited from the treatment (60%) and their treatment goals were met or partly met (66.6%), as measured by evaluations made by the patient or the treatment team. More than two thirds (69.2%) of the participants for whom this information was available remained weight stable or showed an increase in BMI.

**Conclusions:**

This observational study suggests that many individuals with severe and enduring anorexia nervosa may benefit from flexible treatment, aiming at supporting quality of life. The results highlight the importance of coordinating and integrating the treatment of severe and enduring anorexia nervosa and co-occurring psychiatric disorders as well as medical complications. Further research and international dialogue about the how treatment for this vulnerable patient group should best be organized is called for.

*Trial registration* Trial registration number: NCT05708404. Date of registration: 01/23/2023 (retrospectively registered).

## Abstract (plain language summary)

Approximately 20–30% of individuals with the eating disorder anorexia nervosa develop an enduring form of the illness. In this study a newly developed treatment unit for patients with severe and enduring anorexia nervosa was described. The treatment is flexible and aims at enhancing quality of life. Patient characteristics, treatment goals, and course of treatment from the first five years of operation were analyzed. The participants were patients treated at the new treatment unit. All participants were women. The study was based on patient records. On average, the participants had had a diagnosed eating disorder for more than 12 years, and self-reported eating disorder symptoms for 19 years. Many participants had severe eating disorder symptoms, other psychiatric illnesses, extreme underweight, and other medical conditions. Their own treatment goals concerned improving physical health, reducing eating disorder symptoms, improving psychological well-being, and improving quality of life. Many participants benefited from the treatment. This study shows that individuals with severe and enduring anorexia can benefit from flexible treatment that supports quality of life.

## Introduction

Anorexia nervosa (AN) is a serious eating disorder. Approximately 20–30% of people with AN do not recover fully despite treatment but develop an enduring form of the disorder [1, 2]. During recent years, clinicians and researchers have begun to distinguish and pay increasing attention to this patient group. A staging model of AN has been proposed and explored, where different stages of the illness can be distinguished from each other and may require different types of treatment [3, 4]. Severe and enduring anorexia nervosa (SE-AN) has been described using criteria such as an illness duration of ≥ 3–7 years, clinical severity, and having received, but not benefited from, evidence-based treatment [5, 6]. The quality of life of people with SE-AN has been found to be severely impaired [7]. SE-AN is associated with medical and psychiatric comorbidities and complications, and with high mortality [8].

A variety of treatments have been used for SE-AN, with mixed results. Both outpatient, day-hospital and inpatient treatments appear to result in short-term weight increase and symptom reduction [9]. Different drugs and novel biological treatments such as repetitive transcranial magnetic stimulation are also being explored [10, 11]. In the only randomized controlled trial (RCT) of psychotherapeutic treatments for SE-AN, Touyz et al. [12] found that SE-AN patients benefited from both outpatient cognitive behavioral therapy (CBT) and specialist supportive clinical management (SSCM); both treatments were modified to prioritize harm minimization and quality of life. Both CBT and SSCM resulted in positive changes regarding quality of life, mood disorder symptoms, social adjustment, body mass index (BMI), eating disorder symptoms, and motivation for change. In the treatment of SE-AN, it has been proposed that it may be beneficial to place the focus on improving quality of life, medical safety, and overall functioning in the presence of an enduring illness, rather than always focusing on full weight restoration and recovery [4, 12–14]. Recovery from AN can be conceptualized as encompassing both physical recovery (such as weight restoration) as well as behavioral and psychological recovery [15]. In Toronto, Canada, and Stockholm, Sweden, outpatient units for patients with SE-AN who have not benefited from previous eating disorder treatments have been developed. Treatment at these units has been described as aiming at harm-reduction and enhancing quality of life in the presence of an eating disorder [16, 17]. William et al. [17] reported that patients treated at the unit experienced improvements in global distress, hopelessness, eating disorder symptoms and BMI, but not in quality of life. In sum, however, there is a notable paucity of research evidence for treatments of SE-AN, and a need to further develop and improve treatments for this patient group [9]. A need for tailored, flexible treatments that focus on therapeutic alliance and take SE-AN patients’ ambivalence towards treatment and recovery into account has been highlighted in the literature [9]. The limited understanding and paucity of treatments for this patient group has even been described as a crisis in the eating disorders field [10].

At the HUS Helsinki University Hospital Eating Disorder Unit in Helsinki, Finland, a new outpatient unit for patients with SE-AN was established in May 2017. The unit is the first of its kind in Finland. The aim of this study is to describe the first years of operation at this specialized treatment unit: the aims and contents of the treatment, the characteristics of the patients, their treatment goals, as well as the course of their treatment.

## Methods

### Study design

An observational study design was chosen because it enabled observing the elements and course of treatment is a real-world clinical setting, improving ecological validity. The study was a registry study, based on the participants’ medical records. Electronic medical records were gradually taken into use at the HUS Helsinki University Hospital in 2004 and include medical information of treatment taken place within the hospital district. In the planning phase of the study, the first and last author decided on which variables to include and information to collect based on the aims of the study: describing (1) the contents of the treatment, (2) the participants’ eating disorder and psychiatric symptomatology, (3) the participants’ medical status, (4) the participants’ treatment goals, (5) treatment duration and frequency, and (6) how treatment goals were met and whether or not participants benefited from the treatment (as measured by an evaluation made by the participant and/or the treatment team). The selection of variables was also guided by clinical knowledge of the type of information typically recorded in the medical records (for example somatic and psychiatric variables as well as questionnaires used at the eating disorders unit).

### Participants

The participants consisted of all referrals (*N* = 22) resulting in a treatment or assessment period at the unit between May 2017 and May 2022. Two participants were treated twice at the unit: after their treatment at the unit had ended, they were later re-referred and a new assessment was conducted. The total number of individual patients treated was therefore 20. The participants are described in more detail in the Results section.

### Data collection

Information regarding age, gender, illness duration (the age of reported symptom onset and age of formal AN diagnosis), psychiatric comorbidities, lifetime traumatic experiences, substance use, duration of the treatment, number of sessions, and reasons for the treatment ending was gathered from medical records. Information about when the participants were first diagnosed with their eating disorder was missing for 2 (10%) of the individual participants, because they had been ill for a longer time than electronic patient records had been used in the hospital district or first diagnosed in another health care district. Detailed information about all previous treatments was not available, because many participants had been treated in several health care districts in Finland, using different medical record systems, or had started treatment before electronic patient records were used.

Information about the participants’ physical health and medical status at the beginning of treatment was also gathered from patient records: BMI (kg/m^2^), blood pressure, heart rate, bone density, and symptoms related to malnutrition, such as muscle atrophy, dry skin, and amenorrhea.

Eating disorder symptoms (Eating Disorder Examination Questionnaire; EDE-Q) [18], depressive symptoms (BDI; Beck Depression Inventory) [19], psychosocial functioning (CIA; The Clinical Impairment Assessment Questionnaire) [20] were assessed at the beginning of the treatment, and this information was collected from the patient records. The EDE-Q is a 28-item self-report questionnaire that assesses eating disorder symptoms. In the present study, the global EDE-Q score was calculated and used, with a higher score indicating more eating disorder symptoms. The BDI is a 21-item self-report inventory that measures the presence and degree of depression, with a higher total score indicating more severe depression. The CIA is a 16-item self-report measure of psychosocial impairment due to an eating disorder, with a higher total score indicating more impairment. The BDI score was available for 17 (77.3%) participants, the CIA score for 13 (59.1%) participants, and the EDE-Q scores for 11 (50%) participants. At the end of treatment, EDE-Q, BDI, and CIA scores were only available for a small number of participants (*n* = 3–4, 13.6–18.2%); either due to the participant not having been given the questionnaires, the participant not having returned them, or the score not having been recorded in the medical records. The EDE-Q, BDI, and CIA scores at the end of treatment were therefore not included in this study.

In addition, information about the participants’ own treatment goals at the beginning of treatment was gathered from patient records, as well as information about how they had been met at the end of the data collection. Information about whether the participants had benefited from the treatment was also gathered. For 15 (68.2%) of the participants this information was written out in their patient records: either by describing that the participant herself felt that she had or had not been helped by or benefited from the treatment, or as a clinical evaluation made by the treatment team.

### Treatment description

The overarching aims of the treatment were to enhance quality of life, maintain medical stability, and minimize harm for patients with SE-AN. Patients with an illness duration of ≥ 10 years, at least three previous eating disorder treatment attempts, and a stable medical status with a BMI > 12 could be treated at the unit. Contraindications of the treatment were defined as substance addiction, severe personality disorders, acute suicidality or self-harm, and/or other co-occurring severe psychiatric disorders. The requirements of the treatment were that patients’ weight should not decrease during treatment, and that patients should adhere to the plans that have been mutually agreed on during the planning phase. The duration of the treatment was flexible; the treatment did not have a set duration or end date.

Treatment at the unit started with an evaluation and planning phase with the unit’s psychiatrist and registered nurse. The third author functioned as the unit’s psychiatrist (MD, Ph.D.) and has several years of experience working with eating disorder patients. The unit’s nurse has a degree in nursing and likewise several years of experience working with eating disorder patients. During these initial sessions, the participants’ psychological and medical status and eating disorder symptomatology were assessed using interviews, standardized measures (EDE-Q [18], BDI [19], CIA [20], as well as a medical exam conducted by the unit’s psychiatrist (typically assessing height, weight, heart rate, blood pressure, possible muscular atrophy, and other possible symptoms related to malnutrition). Individual treatment goals were set in collaboration with the participant during the evaluation phase. Treatment at the unit was mainly implemented by the unit’s registered nurse. The treatment was largely based on key elements of SSCM: clinical management of eating disorder symptoms and behaviors, psychoeducation, a focus on therapeutic alliance, and patient-driven supportive treatment. In line with SSCM, treatment was based on the unique needs of each participant, with the participant setting the pace for change [21, 22]. The treatment frequency and setting were flexible and implemented according to each participant’s individual needs and wishes. Treatment sessions included sessions at the treatment unit, video and phone sessions, support via text messaging, meetings where participants’ family members were present, as well as meetings in the participants’ own environments (e.g. in their homes, coffee shops, museums, parks, or at the library). Some of the participants (*n* = 6, 27.3%) also participated in CBT-based psychotherapeutic interventions (10–20 sessions) with the first author. Indications of psychotherapeutic interventions at the unit include the patient being motivated to participate in a brief psychotherapeutic intervention, identifying treatment goals suitable for a brief psychotherapeutic intervention, and being well enough to participate in 45-min-long sessions on a regular basis. The first author is a licensed clinical psychologist and psychotherapist, with a Ph.D. in psychology and several years of experience working with eating disorder patients. The goals of the psychotherapeutic interventions were set in collaboration with the participants. Possible goals of psychotherapeutic interventions at the unit include, for example, supporting emotion regulation, enhancing motivation for change, or targeting maladaptive and inflexible habits. Participants treated at the unit met with unit’s psychiatrist at least once a year. A social worker and registered dietician were consulted when indicated. The social worker can for example be consulted when patients need assistance with social assistance and benefits. The registered dietician can for example be consulted when patients have questions about their diet or need support managing co-occurring medical symptoms (e.g. food intolerance or gastrointestinal conditions).

### Data analysis

Descriptive statistics were analyzed using IBM SPSS Statistics (Version 25) [23]. Treatment goals were categorized using inductive content analysis [24] of information gathered from patient records. The data was collated and grouped into categories, and similar categories were further combined into higher order categories. The analyses were conducted by the first author and independently reviewed by the second author.

### Research ethics

Study permission, research permission, and ethics approval (ethics approval number HUS/1966/2019) were obtained by the HUS Helsinki University Hospital and its ethics committee before the beginning of the data collection.

## Results

### Participant characteristics and symptomatology

There were 22 sets of results and 20 unique participants, as two participants were treated twice at the unit. All participants were women. The mean age of the participants when starting treatment at the unit was 35.86 years (*SD* = 10.28, range 24–61 years). On average, the participants had had a diagnosed eating disorder for 12.80 years (*SD* = 7.90, range 4–37 years), and self-reported eating disorder symptoms for 19.04 years (*SD* = 10.80, range 5–47 years) when starting the treatment. All participants except two had a AN diagnosis; two (9.1%) participants had a current diagnosis of atypical AN when starting treatment at the unit. These participants presented with AN symptomatology and a history of AN, but a current BMI within the normal range. All participants had received different kinds of previous treatments for their eating disorder and co-occurring psychiatric symptoms for several years. Of the participants 16 (72.7%) had previously received inpatient and/or day-hospital treatment for their eating disorder, all of them on more than one occasion. Of the participants, 9 (40.9%) had previously received manualized CBT for eating disorders (CBT-E) [25] and participated in at least part of the treatment. In addition, many participants had received other types of psychotherapy (lasting from 20 sessions to several years), so that in total, 19 (86.4%) of the participants had previously received some type of psychotherapeutic treatment (most commonly CBT, CBT-E, integrative psychotherapy, or psychodynamic therapy).

At the beginning of treatment at the unit, almost two thirds of the participants reported compulsive exercise, and almost a third reported binge eating or vomiting. A majority had co-occurring psychiatric diagnoses, most commonly depression. Traumatic experiences were mentioned in more than every fifth participant’s records; most commonly developmental trauma in close relationships. The average number of co-occurring psychiatric diagnoses was 1.64 (*SD* = 1.18, range 0–4). The mean BDI score of the participants (*n* = 17) at the beginning of treatment was 27.29 (range 11–44, *SD* = 11.85), indicating moderate depression. The mean CIA global score of the participants (*n* = 13) was 29.38 (range 9–41, *SD* = 8.15), which is comparable to scores from other Nordic patients with diagnosed eating disorders [26, 27]. The mean global EDE-Q score of the participants (*n* = 11) was 3.34 (range 0.91–5.68, *SD* = 1.56) which is likewise comparable to other patients with diagnosed eating disorders [27]. The participants’ eating disorder and psychiatric symptomatology is outlined in Table 1.

**Table 1:** Eating disorder and psychiatric symptomatology at the beginning of treatment

	Yes, *n* (%)	No, *n* (%)
Self-induced vomiting	7 (31.8)	15 (68.2)
Binge-eating	7 (31.8)	15 (68.2)
Compulsive exercise	15 (68.2)	7 (31.8)
Substance misuse	2 (9.1)	20 (90.9)
Traumatic experiences	5 (22.7)	17 (77.3)
*Co-occurring psychiatric diagnoses*	17 (77.3)	5 (22.7)
Depression	14 (63.6)	8 (36.4)
Anxiety	7 (31.8)	15 (68.2)
Obsessive–compulsive disorder	9 (40.9)	13 (59.1)
Any personality disorder	5 (22.7)	17 (77.3)
Emotionally unstable personality disorder	4 (18.2)	18 (81.8)

To protect the participants’ privacy, psychiatric diagnoses that only one participant had were excluded

The mean BMI of the participants at the beginning of treatment was 14.95 (*SD* = 2.80), indicating extreme AN [28]. A notable majority of the participants, for whom information about their medical status was available, had muscular atrophy, amenorrhea, and osteopenia or osteoporosis. Hypertension was reported in every fourth participant, and bradycardia in almost every third. In addition, for 11 participants (50%) dry skin was reported, for two participants (9.1%) abnormal laboratory findings (e.g. leukopenia or hyponatremia) were reported, for one participant (4.5%) cyanosis was reported, and for one participant (4.5%) lanugo hair was reported. The medical condition of the participants at the beginning of treatment is described in Table 2.

**Table 2:** Medical condition at the beginning of treatment

	Yes, *n* (%)	No, *n* (%)	Missing data, *n* (%)
Hypertension (> 120/80 mmHg)	5 (26.3)	14 (73.7)	3 (13.6)
Hypotension (< 90/60 mmHg)	1 (5.3)	18 (94.7)	3 (13.6)
Bradycardia (heart rate < 60 BPM)	6 (31.6)	13 (68.4)	3 (13.6)
Osteoporosis or osteopenia	17 (85)	3 (15)	2 (9.1)
Muscular atrophy	13 (68.4)	6 (31.6)	3 (13.6)
Amenorrhea	17 (81)	4 (19)	1 (4.5)
	Range	*M (SD)*	
BMI	11.60–22.10	14.95 (2.80)	1(4.5)

*BPM* Beats per minute. The descriptive analyses were based on available data

### Treatment goals

The treatment goals at the unit were flexible, individualized, and agreed on together with the participant at the beginning of the treatment. For 17 participants (77.3%), clear treatment goals were described in their patient records. For five (22.7%) of the participants, including one of the two participants who had two separate treatment periods at the unit, the assessment or treatment period was so brief that no clear goals were described. The most common treatment goals concerned improving physical health and reducing eating disorder symptoms, improving psychological well-being, and improving quality of life. Two participants mentioned recovery from the eating disorder as a treatment goal. The treatment goals of the participants are outlined in Table 3.

**Table 3:** Treatment goals of the participants at the unit

Theme	*n* (*%*)	Treatment goal	*n* (*%*)
Recovery	2 (11.8)	Recovery from the eating disorder	2 (11.8)
Eating disorder symptoms and physical health	11 (64.7)	Increasing and improving eating and energy intake	5 (29.4)
		Better physical health and weight gain	4 (23.5)
		Following up physical health and avoiding worsening of one’s physical status	4 (23.5)
		Decreased eating disorder symptoms	3 (17.6)
		Increasing strength and energy	3 (17.6)
Psychological well-being	5 (29.4)	Improving and supporting psychological well-being	4 (23.5)
		Managing psychiatric co-morbidities	2 (11.8)
Daily living and quality of life	12 (70.6)	More positive content in one’s daily life	4 (23.5)
		Improving functional ability and independence through supporting physical health	4 (23.5)
		Improving social functioning and maintaining interpersonal relationships	3 (17.6)
		More relaxation and rest	2 (11.8)
Other	2 (11.8)	Planning and arranging further treatment	1 (5.9)

This table is based on 17/22 (77.3%) of the participants. For 5/22 (22.7%) of the participants, no clear treatment goals were formulated. One participant may have several treatment goals, also within the same theme. One treatment goal from the last category was excluded to protect the participant’s privacy

### Treatment course

The duration of treatment varied between 1 and 59 months (*M* = 15.4, *SD* = 17.5). For 7 participants (31.8%) the treatment period consisted of ≤ 5 sessions. These were mainly assessment and/or single supportive sessions. Even a brief contact with the unit aimed at including supportive elements such as psychoeducation, managing symptoms, and focusing on alliance. At the end of the data collection, the average number of treatment sessions or contact with the treatment unit was 28.18 (range 2–112, *SD* = 29.06). This number included sessions at the treatment unit, phone and video sessions, meetings in the participants’ own environments and supporting participants and answering their questions via text messaging. At the end of the data collection, five participants’ (22.7%) treatment was still ongoing. Information about whether the participant had benefited from the treatment was available for 15 (68.2%) participants; recorded in the patient records as a description of the participant’s own evaluation of the treatment benefits and/or a clinical evaluation made by the team. Of these, 9 (60%) were evaluated to have benefited from the treatment and 6 (40%) to not have benefited from it. For seven participants (31.8%) it could not be concluded from patient records whether they had benefited from the treatment or not. Information about whether one’s treatment goals had been met was available for 15 (68.2%) participants. For five of them (33.3%) the treatment goals had been met, for five participants (33.3%) they had not been met, and for five participants (33.3%) they had been partly met. Information about BMI at both the beginning of treatment as well as the end of treatment, or the end of the data collection for participants whose treatment continued, was available for 13 participants (59.1%). For these participants the mean BMI at the beginning of treatment was 14.95 (*SD* = 3.30, range 11.60–22.10), indicating extreme AN. Their mean BMI at the end of treatment/the data collection was 15.60 (*SD* = 2.63, range 11.40–19.40), indicating severe AN [28]. For four (30.8%) of these 13 participants, BMI was lower at the end of treatment, while 9 (69.2%) participants remained weight stable or had an increased BMI at the end of the treatment/data collection.

For the 17 participants (77.3%) whose treatment had ended by the end of the data collection, the most common reason for treatment being discontinued (*n* = 6, 35.3%) was that the participant needed more intensive psychiatric treatment for co-occurring psychiatric problems than could be provided at the unit. For example, severe mood or anxiety disorder symptoms cannot be sufficiently treated at the unit. Other reasons for treatment being discontinued included not finding the treatment useful or necessary (*n* = 3, 17.6%), finding the treatment too burdening (*n* = 2, 11.8%), or having benefited so much from the treatment that the participant no longer felt a need for it (*n* = 2, 11.8%). Reasons for treatment being discontinued that only encompassed one participant have been excluded to protect the participants’ anonymity.

## Discussion

In the present study, a newly developed treatment unit for patients with SE-AN was described. The aims and contents of the treatment were described, and patient characteristics, treatment goals, and course of treatment from the first five years of operation were analyzed. The treatment model was flexible, patient-centered, and aimed at enhancing quality of life, maintaining medical stability, and minimizing harm for patients with SE-AN. The treatment was mainly based on key elements of SSCM and tailored to every patient’s unique needs and treatment goals. In contrast with a previously described SSCM treatment for SE-AN [12], treatment at the unit was flexible regarding treatment duration, frequency, and setting. The treatment had similarities with previously described outpatient units for SE-AN in Sweden and Canada, aiming at improving quality of life in the presence of an eating disorder [16, 17]. In sum, the present treatment unit is one of the first of its kind to be systematically described.

On average, the participants had had self-reported eating disorder symptoms for 19 years, and diagnosed AN for almost 13 years, when being referred to the unit. It can be noted that the latency between first self-reported symptoms and being diagnosed with and treated for AN in some cases was several years long. This could possibly have played a part in the eating disorder taking on an enduring form. The participants’ symptomatology commonly included psychiatric comorbidities, compulsive exercise, self-induced vomiting, binge eating, and extreme underweight. Many participants presented with medical conditions such as amenorrhea, osteopenia or osteoporosis, muscle atrophy, dry skin, hypertension, or bradycardia. These medical complications are due to malnutrition and can occur in AN and SE-AN [29, 30]. In comparison to a previous study of AN patients, the prevalence of hypertension was elevated [31]. In sum, these results show that the participants, on average, were severely ill. A notable majority of the participants had previously received several inpatient and/or day hospital treatments for their eating disorder, as well as psychotherapeutic treatment.

The aims of the treatment model were reflected in the treatment goals of the participants. Most treatment goals concerned improving physical health and reducing eating disorder symptoms, improving psychological well-being, and improving daily living and quality of life. For two participants, full recovery was mentioned as a treatment goal. At the end of treatment or the data collection, the majority of participants for whom this information was available, was evaluated to have benefited from the treatment, and their treatment goals had been met or partly met. More than two thirds of the participants for whom this information was available remained weight stable or showed an increase in BMI, in line with the overarching aim of the treatment to maintain medical stability. At the end of treatment or the data collection, the mean BMI of the participants was slightly higher and indicated severe AN [28], as compared to at the beginning of treatment, when mean BMI indicated extreme AN [28]. However, this comparison should be interpreted with caution, because information about BMI at both the beginning and the end of treatment/data collection was only available for 13 (59.1%) of the participants, and the numbers were too small for inferential statistical analyses. For several participants, treatment at the unit was discontinued because of a need for more intensive treatment for their psychiatric comorbidities. It should also be noted that some participants did not find the treatment model beneficial. Some participants also expressed finding treatment in general too burdening, which possibly may reflect experiences of many years of different types of eating disorder treatments.

### Clinical implications

For clinicians planning and implementing treatment for individuals with SE-AN, the present findings emphasize the importance of a truly patient-centered approach regarding both the timing, goals, contents, setting, and intensity of the treatment. They also highlight the importance of collaboration between different treatment units, both psychiatric and physical. Wonderlich et al. [10] have likewise pointed out the importance of identifying comorbid disorders and the most significant problems and prioritize treatment strategies for this patient group accordingly. The complex care needs of this patient group, both psychiatric and medical, require clinical expertise and co-ordination with other services.

It has been argued that treatment for patients with SE-AN should not solely focus on harm reduction and quality of life, as recovery is possible even after decades of illness [2]. The possibility of physical, behavioral, and psychological recovery as well as maintaining hope is a very important perspective when working with patients with SE-AN. We would, however, argue that we must meet our patients where they are. Focusing on patients’ own treatment goals and enhancing quality of life does not equal giving up hope of either symptom reduction, improved well-being, or even full recovery. However, in the face of severe and enduring illness, after several years of eating disorder treatments that have not yet led to recovery, factors such as treatment burnout, deep ambivalence about recovery, and a sense of powerlessness and loss of hope may be present [10, 14]. These factors must be considered and addressed when making decisions about when to strive for full weight restoration and recovery. In line with Russell et al. [14] we would argue that we need to find a balance between striving for fuller recovery, without frightening our patients with SE-AN away from treatment. What this balance looks like is likely to be different for each unique patient. In our experience, the challenge lies in maintaining a truly patient-centered approach, while striving to both utilize and develop evidence-based treatment models for this vulnerable patient group.

Based on the present results and our clinical experience, we suggest that possible fruitful aspects of such treatment models include a multiprofessional team, formulating unique treatment goals together with the patient, approaching change in each patient’s own pace, and flexibility regarding the duration, setting, and frequency of the treatment. It is also recommended that the treatment setting allows for the management and treatment of co-occurring psychiatric and medical conditions and symptoms, and/or referring patients to adequate treatment of these. It is important that the team that is planning, coordinating, and implementing the treatment have adequate training and experience in treating eating disorders. We also recommend assessing the effects of treatment with the help of medical exams and standardized measures of eating disorders symptoms, psychiatric symptoms, and quality of life.

### Strengths and limitations

A key strength of this study was its novelty: it described the first five years of operation at a new treatment unit for SE-AN. It’s also one of very few studies to systematically describe the clinical characteristics of patients with SE-AN presenting for treatment. A main limitation of the study was that part of the data was missing: in particular standardized measures of eating disorder symptoms and depression at the end of treatment. The COVID-19 pandemic unfortunately disrupted the data collection, as some treatment sessions were cancelled, and others were carried out as video sessions. An additional limitation is that no standardized measures of quality of life were included. For some of the participants, it could also not be concluded from patient records whether they had benefited from the treatment or not, and whether their treatment goals had been met. Taken together, these factors only allow us to draw tentative conclusions about the benefits and effects of this type of treatment, and further research on the topic is called for. It should also be mentioned that the first and third author were involved in delivering the treatment, which potentially could be a source of bias when interpreting the results. It is also worth noting that the participants, on average, were severely ill. It is therefore likely that the results of the study are not generalizable to all individuals with enduring eating disorders.

### Conclusions and future directions

In sum, this observational study suggests that many individuals treated for SE-AN may benefit from flexible treatment, primarily aiming at supporting quality of life. This conclusion is drawn based on the participants’ as well as the clinicians’ evaluations of how beneficial the present treatment was, as well as how well the treatment goals of the participants were met. It can also be noted that more than two thirds of the participants for whom this information was available remained weight stable or displayed an increase in BMI during the treatment. Further research using more standardized measures of eating disorder symptomatology and quality of life is needed to further test this tentative conclusion, preferably through RCTs. To better understand the treatment needs and experiences of this patient group qualitative studies are also needed. In addition, further research and international dialogue about the how treatment for this vulnerable patient group and their diverse needs should best be organized is called for.

## Data Availability

The dataset generated during the current study is not publicly available due to privacy restrictions. Anonymized data that support the findings of this study are available upon reasonable request from the corresponding author (MÅ).

## Abbreviations

AN: Anorexia nervosa
BDI: Beck Depression Inventory
BMI: Body mass index
CBT: Cognitive behavioral therapy
CIA: The Clinical Impairment Assessment Questionnaire
EDE-Q: Eating Disorder Examination Questionnaire
RCT: Randomized controlled trial
SE-AN: Severe and enduring anorexia nervosa
SSCM: Specialist supportive clinical management
